# Obituary

**Published:** 1956-10

**Authors:** 


### *Obituary*

Arthur Harold Gale died on 12th October at the age of 55. He had been in poor health for months, with several periods in hospital.

He qualified from U.C.H. in 1926; he gained the D.P.H. in 1930 and M.D. Oxon., in 1933. In 1944 his Milroy Lectures at the Royal College of Physicians were entitled "A century of changes in the mortality and incidence of the principal infections which cause death or disability in childhood". Before he came to Bristol Dr. Gale was a senior medical officer in the Ministry of Health and medical officer in the Ministry of Education. In 1950 he was appointed Director of Postgraduate Medical Studies at the University and organized the popular series of Sunday lectures for family doctors.

Much of his work was unobtrusive and little recognized; for example, few realize that it was Gale who edited the *Guide to Antibiotics*, a booklet that was published by the Bristol Royal Infirmary and received a lauditory annotation in the *Lancet*. His scholarly and restrained outlook is reflected in his published researches into epidemiology.

As Assistant Editor of this journal, Dr. Gale saw into print all the facts and details in Notes and News; he undertook much sub-editing and added his own contributions in annotations and original articles.

He was a gentle and kind man and he will be sadly missed by many.

*E. R. H. writes:*

In the hurry and bustle of post-war life one still occasionally meets the man who makes time to put aside his own work to help others, to recognize and discuss their problems without the feeling that there is something more pressing deserving his attention.

Such an individual was Arthur Gale. Nothing was too much trouble to him if it would help another on his way.

His all-too-infrequent visits were a delight to the household, particularly the children, into whose life he was fully admitted.

Perhaps what we remember most was his courage. Fully aware of the seriousness of his illness, he continued to make light of it to the last and refused to allow consideration of his health to interfere with his duties.

